# The Insensitivity of TASK-3 K_2_P Channels to External Tetraethylammonium (TEA) Partially Depends on the Cap Structure

**DOI:** 10.3390/ijms19082437

**Published:** 2018-08-18

**Authors:** Guierdy Concha, Daniel Bustos, Rafael Zúñiga, Marcelo A. Catalán, Leandro Zúñiga

**Affiliations:** 1 Centro de Investigaciones Médicas (CIM), Programa de Investigación Asociativa en Cáncer Gástrico (PIA-CG), Escuela de Medicina, Universidad de Talca, Talca 3460000, Chile; guierdy@gmail.com (G.C.); danielbustos.ibi@gmail.com (D.B.); rafaelzunigah@gmail.com (R.Z.); 2 Laboratorio de Fisiología Epitelial, Facultad de Ciencias de la Salud, Universidad Arturo Prat, Iquique 1130000, Chile; macatalan@unap.cl

**Keywords:** TASK-3 channels, two-pore domain channel, TEA

## Abstract

Two-pore domain K^+^ channels (K_2_P) display a characteristic extracellular cap structure formed by two M1-P1 linkers, the functional role of which is poorly understood. It has been proposed that the presence of the cap explains the insensitivity of K_2_P channels to several K^+^ channel blockers including tetraethylammonium (TEA). We have explored this hypothesis using mutagenesis and functional analysis, followed by molecular simulations. Our results show that the deletion of the cap structure of TASK-3 (TWIK-related acid-sensitive K^+^ channel) generates a TEA-sensitive channel with an IC_50_ of 11.8 ± 0.4 mM. The enhanced sensitivity to TEA displayed by the cap-less channel is also explained by the presence of an extra tyrosine residue at position 99. These results were corroborated by molecular simulation analysis, which shows an increased stability in the binding of TEA to the cap-less channel when a ring of four tyrosine is present at the external entrance of the permeation pathway. Consistently, Y99A or Y205A single-residue mutants generated in a cap-less channel backbone resulted in TASK-3 channels with low affinity to external TEA.

## 1. Introduction

Leak K^+^ channel family, also known as K_2_P or two-pore domain K^+^ channels, are widely expressed among different cell types, where they play a critical role in setting the resting membrane potential [1,2]. The K_2_P family consists of fifteen different members divided into six subfamilies based on structural and functional properties [3,4,5,6]. In humans, K_2_P channels are encoded by the *KCNK* gene family and mutations of its genes have been associated with several pathologies. For instance, TASK-1 malfunction is linked to pulmonary hypertension [7] and cardiac arrhythmias [8]. Additionally, mutations of TASK-3 are associated with Birk Barel syndrome [9], and TASK-3 overexpression was found in human breast cancer tumors, where it has been proposed to act as a proto-oncogene [10]. Further study showed that TASK-3 gene knock down in breast cancer cells is associated with an induction of cellular senescence and cell cycle arrest [11].

Regarding protein structure, each K_2_P channel subunit has four transmembrane domains (TM1-TM4) and two pore-forming domains (P1 and P2). Therefore, two subunits are required to form a functional channel [12,13]. 

Recently, X-ray crystallographic structures of TRAAK (TWIK-related arachidonic acid-stimulated K^+^ channel), TREK1 (TWIK-Related K^+^ Channel), TREK2 and TWIK-1 (Tandem pore domains in a weak inward rectifying K^+^ channel) channels have been reported, giving important insights into the K_2_P channel function [14,15,16,17]. Structural studies revealed that K_2_P channels display an exclusive extracellular cap domain formed by the extracellular loop that connects the first transmembrane domain and the first pore-forming sequence (TM1-P1 loop). The cap domain forms two tunnel-like side portals, known as the extracellular ion pathway (EIP) [18]. Also, the cap structure has been proposed as a barrier that hinders the access of classical K^+^ channel blockers to their binding sites. Thus, the cap domain has been proposed to be responsible for the poor sensitivity of K_2_P channels to classical K^+^ channel blockers [15,16]. 

By using mutagenesis, electrophysiology and computational analysis, we herein explored the role of the cap structure and potential residues in the blockade of TASK-3 channel by tetraethylammonium (TEA). 

Our results confirm that the cap structure limits the access of TEA to the binding site in the TASK-3 channel. The deletion of the cap domain (by replacing the Loop1-P1 with a second Loop2-P2), generates a TEA-sensitive TASK-3/2loop2 channel. This TEA sensitivity is explained by a four-tyrosine ring at the mouth of the pore (Y99 and Y205). When the Y99 and the Y205 residues were mutated to alanine in the background of the TASK-3/2loop2, the channels displayed a substantial insensitivity to TEA similar to that observed in wild-type TASK-3 channels. 

## 2. Results

### 2.1. TEA Is a Potent Blocker of Kv2.1 Channel but Not an Effective Blocker of TASK-3 Channel

**Figure 1:**
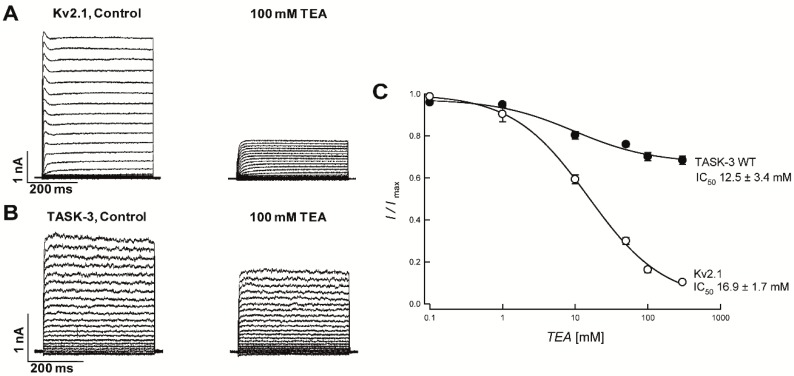
Effect of TEA on Kv2.1 and TASK-3 channels expressed in HEK-293 cells. (**A**) Rat Kv2.1 channel currents measured with a voltage protocol (400-ms steps from −100 mV to 100 mV with an increment of 10 mV and a holding potential of −80 mV) before (left) and after (right) the exposition to 100 mM of TEA. (**B**) Application of 100 mM of TEA on TASK-3 channels, before (left) and after (right) the treatment. (**C**) Dose-response relationship of TEA on Kv2.1 and TASK-3 channel. TEA blockade was analyzed at +80 mV test pulse. Kv2.1 IC_50_ value was 16.9 ± 1.7 mM (*n* = 4).

We first examined the effect of external TEA on Kv2.1 (a member of the voltage-dependent potassium channels family) and TASK-3 channels (member of the K_2_P channel family) expressed in HEK-293 cells. We found that the application of 100 mM TEA led to a strong inhibition of Kv2.1 currents (~85%) (Figure 1A), with an IC_50_ value of 16.9 ± 1.7 mM at +80 mV (Figure 1C). In contrast, the blockade of TASK-3 by 100 mM TEA was very low (IC_50_ value of 12.5 ± 3.4 at 80 mV), reaching 30% inhibition at saturating TEA concentrations at +80 mV (Figure 1B,C), consistent with previously reported findings [5,19].

**Figure 2:**
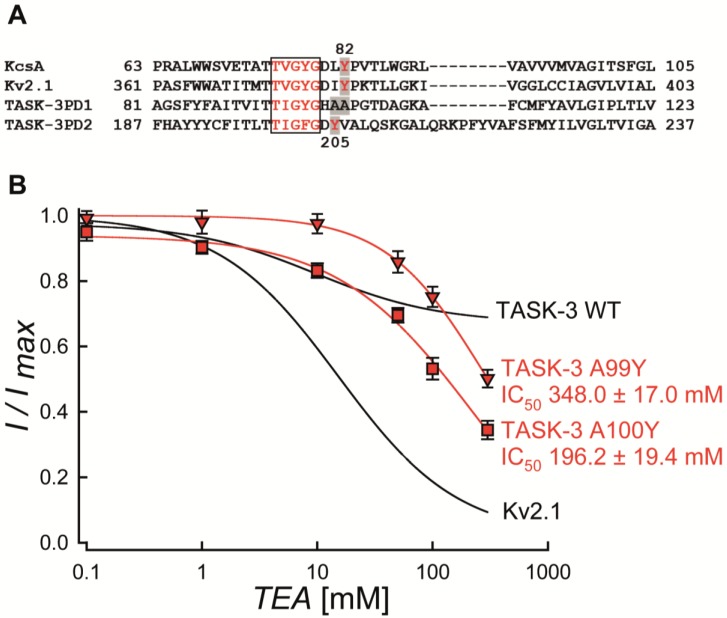
Multiple alignment of selected K^+^ channel pore domains and effect of TEA on TASK-3 and Kv2.1 mutant channels expressed in HEK-293 cells. (**A**) Amino acid sequence alignment of KcsA, Kv2.1 and TASK-3 pore domains. Gaps are indicated by dashes, letters with gray background are the residues implicated in the TEA binding site (Y82, Y380, A99, A100 and Y205, respectively). The selectivity filter signatures are boxed and the numbers are indicated. The PD1 and PD2 signify pore domains 1 and 2 of TASK-3 channels, respectively. (**B**) Dose-response curve of TEA on A99Y (red triangle) and A100Y (red square) mutants. The block was analyzed at the end of the test pulse at +80 mV. Results are shown as means ± SEM. The black lines were taken from the fits in Figure 1C and correspond to the TEA inhibition curves for TASK-3 WT and Kv2.1, respectively.

The high affinity of TEA for Kv or Kir channels depends on the presence of aromatic residues (Tyrosine or Phenylalanine) at the mouth of the pore [20,21,22,23]. For instance, it has been reported that residues Y82 and Y380 are key residues involved in TEA-mediated blockade in KcsA and Kv2.1 channels, respectively (see Figure 2A) [20,21,22,23]. 

Taking advantage of the availability of Kv2.1-containing expression vector, we mutated the residue Y380 for alanine (Y380A) in Kv2.1 channels and found an important increase in the IC_50_ value (~3-fold, IC_50_ 55.5 ± 2.2 mM) in the Y380A mutant (Figure S1). This finding is consistent with the Y380 residue playing a key role in the sensitivity of Kv2.1 to TEA, as previously reported [24,25].

We then examined the alignment of the pore domains of KcsA, Kv2.1 and TASK-3 (each P domain, separately) (Figure 2A). The A100 residue in the first pore region of TASK-3 (P1-domain) is the equivalent amino acid to Y82 (KcsA) and Y380 (Kv2.1). In contrast, the second pore region of TASK-3 (P2-domain) displays a tyrosine residue in position 205 (Y205) (Figure 2A). Thus, the presence of only one tyrosine (Y205) placed in the P2-domain per TASK-3 subunit (two tyrosine for a functional dimeric channel), should explain the extracellular TEA insensitivity obtained in the TASK-3 channel. To test this possibility, the single mutation A100Y on the WT background was investigated. As shown in Figure 2B, this mutant was poorly TEA-sensitive and had an IC_50_ value of 196.2 ± 19.4 mM (*n* = 4). To rule out a possible insensitivity to TEA due to a higher distance between the blocker and the selectivity filter compared to that existing in TEA-sensitive channels, we also evaluated the single mutation A99Y on the TASK-3 background that showed a sensitivity to TEA similar to that observed in the A100Y mutant (IC_50_ value of 348.0 ± 17.0 mM; *n* = 3) (Figure 2B). 

### 2.2. Cap Structure Deletion in TASK-3 Generates Poorly Selective Channels

The low sensitivity of TASK-3 channels to TEA has been explained by the presence of the cap structure, which blocks the access of TEA to its binding sites [15,16]. 

**Figure 3:**
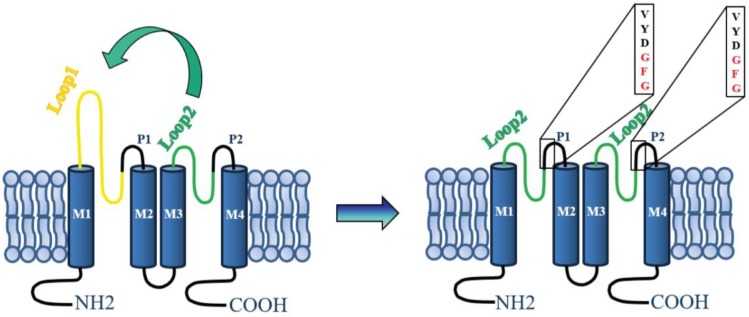
Schematic representation of the TASK-3 channel construct. A representation of the TASK-3 WT channel topological model is shown at the left. In this representation, each subunit has two pore-forming domains (P loops) and four transmembrane domains (denoted M1-M4). To the right is shown the TASK-3/2Loop2 channel construct with the amino acid sequence of selectivity filter illustrated in boxes.

To probe the hypothesis proposed for the role of the cap structure in the insensitivity of K_2_P channels to TEA, we constructed TASK-3 channels that lacked the cap structure. This goal was achieved by constructing a cDNA encoding for TASK-3 channels where the cap-forming loop1-P1 sequence was replaced with a loop2-P2 (TASK-3/2loop2) (Figure 3). Therefore, the cDNA encoding for cap-less TASK-3 channels is the one that has two loop2-P2 (TASK-3/2loop2) as external linkers (Figure 3).

**Figure 4:**
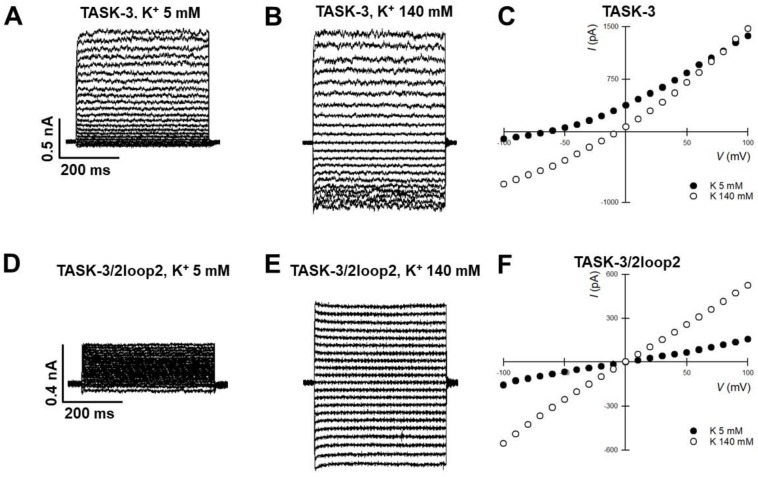
Functional evaluation of TASK-3/2Loop2 construct: effect on rectification, time-dependence and selectivity. Representative whole-cell recordings of TASK-3 (**A**,**B**, *n* = 6) and TASK-3/2Loop2 (**D**,**E**, *n* = 6) K^+^-mediated currents recorded according to the voltage protocol described in the legend of Figure 1. The results in (**A**–**C**) correspond to TASK-3 WT channels and those in (**D**–**F**) to TASK-3/2loop2 construct. In (**A**,**D**), the extracellular medium was 135 mM Na^+^ and 5 mM K^+^. In (**B**,**E**), the extracellular Na^+^ was replaced by an equimolar amount of K^+^(140 mM). (**C**,**F**) shows the representative current-voltage relations for TASK-3 and 2loop2 channels under different extracellular K^+^ concentrations (closed circles 5 mM; open circles 140 mM).

Figure 4A–F shows a comparison of the currents generated by TASK-3 (WT) and TASK-3/2loop2, in physiological (Figure 4A,D) and high external K^+^ concentrations (Figure 4B,E), respectively. TASK-3 WT channels show a characteristic leak potassium current with a normal time dependence and selectivity of K^+^ over Na^+^ (Figure 4A,B), as seen in the current-voltage relations (Figure 4C). Although the TASK-3/2loop2 construct could be readily over-expressed in HEK-293 cells, the magnitude of the currents was lower than those displayed by TASK-3 WT channels (Figure 4D) and showed poor selectivity of K^+^ over Na^+^ when evaluated under physiological conditions (145 mM vs. 5 mM, intracellular vs. extracellular [K^+^]) (Figure 4F). However, robust currents were obtained under symmetrical potassium conditions (140 mM K^+^) (Figure 4E,F). The lack of selectivity displayed by the TASK-3/2loop2 channel might be a consequence of mutating the GYG (Glycine-Tyrosine-Glycine) triplet from the pore forming region 1 to the GFG (Glycine-Phenylalanine-Glycine) triplet from the pore-forming region 2.

### 2.3. A Ring of Four Tyrosines, at the Mouth of the Pore, Confers TEA Sensitivity to TASK-3

We evaluated the effect of TEA blocker on the cap-less TASK-3/2loop2 construct. This construct generates a channel with one tyrosine per P-domain (therefore, four tyrosine per dimeric channel). In this case, a strong sensitivity to the extracellular TEA blockade is expected. 

**Figure 5:**
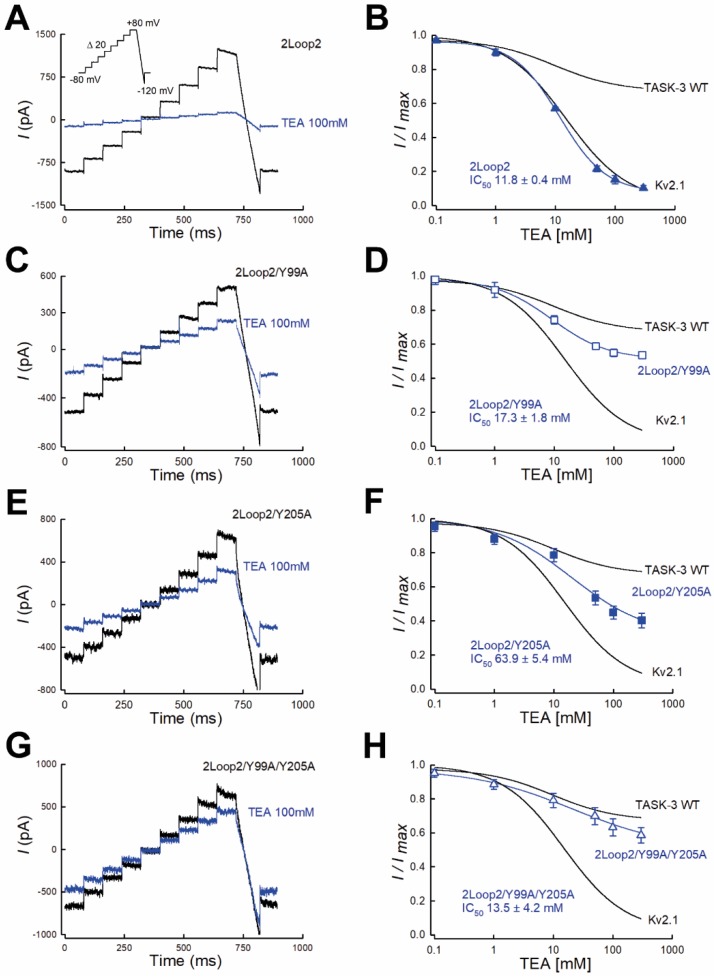
The absence of the cap structure and a ring of four tyrosines confer full sensitivity of TASK-3 channels to TEA. (**A**,**C**,**E**,**G**), whole cell recordings showing the effect of TEA (100 mM) on TASK-3/2loop2 channels. (**A**) TASK-3/2loop2/Y99A channels (**C**) TASK-3/2loop2/Y205A channels (**E**) and TASK-3/2loop2/Y99A/Y205A channels (**G**). (**B**,**D**,**F**,**H**), the graph shows the dose-response curves for extracellular TEA blockade displayed by TASK-3 channels constructs. (**B**) TEA inhibition curves of TASK-3/2loop2 (*n* = 4), TASK-3/2loop2/Y99A (**D**, *n* = 4), TASK-3/2loop2/Y205A (**E**, *n* = 3) and TASK-3/2loop2/Y99A/Y205A (**H**, *n* = 3) mutant channels. Results are shown as means ± SEM. Curves are fits to a 4-parameter logistic function and were constructed by using the average of fitted parameters of the individual experiments. The lines without points are taken from the fits shown in Figure 1C and correspond to the TEA inhibition curves for TASK-3 WT and Kv2.1, respectively.

Indeed, Figure 5A–B shows that cap-less TASK-3 channel was TEA-sensitive and had a maximum inhibition of 90% and an IC_50_ value of 11.8 ± 0.4 mM (*n* = 4) when assayed in symmetrical K^+^ conditions (Figure 5B). 

Given that the activity of the cap-less channel was only detected when recorded under high external K^+^ concentration, we were forced to add TEA without reducing the external K^+^ concentration, thus creating a substantial change in external osmolality. To rule out any possible effect on TASK-3 and Kv2.1 due to a change in external osmolality, we tested the currents displayed by TASK-3 and Kv2.1 channels in response to different external solutions when the osmolality was increased by adding mannitol instead of TEA. As seen in Figure S2, channel activity of both TASK-3 and Kv2.1 was poorly decreased when switched from isosmotic to hyperosmotic solution (800 mOsm). 

To test the possibility that residue Y99 confers, at least in part, the sensitivity of the TASK-3/2loop2 construct to TEA, we mutated residue Y99 for an alanine residue (Y99A) in the backbone of the cap-less TASK-3 channel (TASK-3/2loop2/Y99A). 

As shown in Figure 5C,D, theTASK-3/2loop2/Y99A mutant displayed a partial TEA sensitivity with a maximal inhibition of 46% and an IC_50_ value of 17.3 ± 1.8 mM. By analogy, we also tested the contribution of the Y205 residue of TASK-3 to the TEA sensitivity. Replacement of Y205 for an alanine residue (Y205A) on the background of the mutant TASK-3/2loop2 (TASK-3/2loop2/Y205A) showed a similar pattern to that obtained with the TASK-3/2loop2/Y99A mutant (Figure 5E,F). TASK-3/2loop2/Y205A mutant presented a maximal inhibition of 59% and had an IC_50_ value of 63.9 ± 5.4 mM (Figure 5F).

We then generated a cap-less TASK-3 channel with no tyrosine residues near the pore region (TASK-3/2loop2/Y99A/Y205A mutant) to test its sensitivity to TEA. As shown in Figure 5G,H, mutant channels were essentially insensitive to TEA blockade, with a similar insensitivity to that displayed by the TASK-3 WT channel (Figure 5G,H). Taken together, our data clearly show that, in the absence of the cap structure, TASK-3 channel requires a four-tyrosine ring at the mouth of the pore to be fully blocked by extracellular TEA ions. Therefore, our results are consistent with a partial role of the cap structure to the access of TEA blocker.

### 2.4. Computational Analysis of Extracellular TEA Binding in TASK-3 Channel

**Figure 6:**
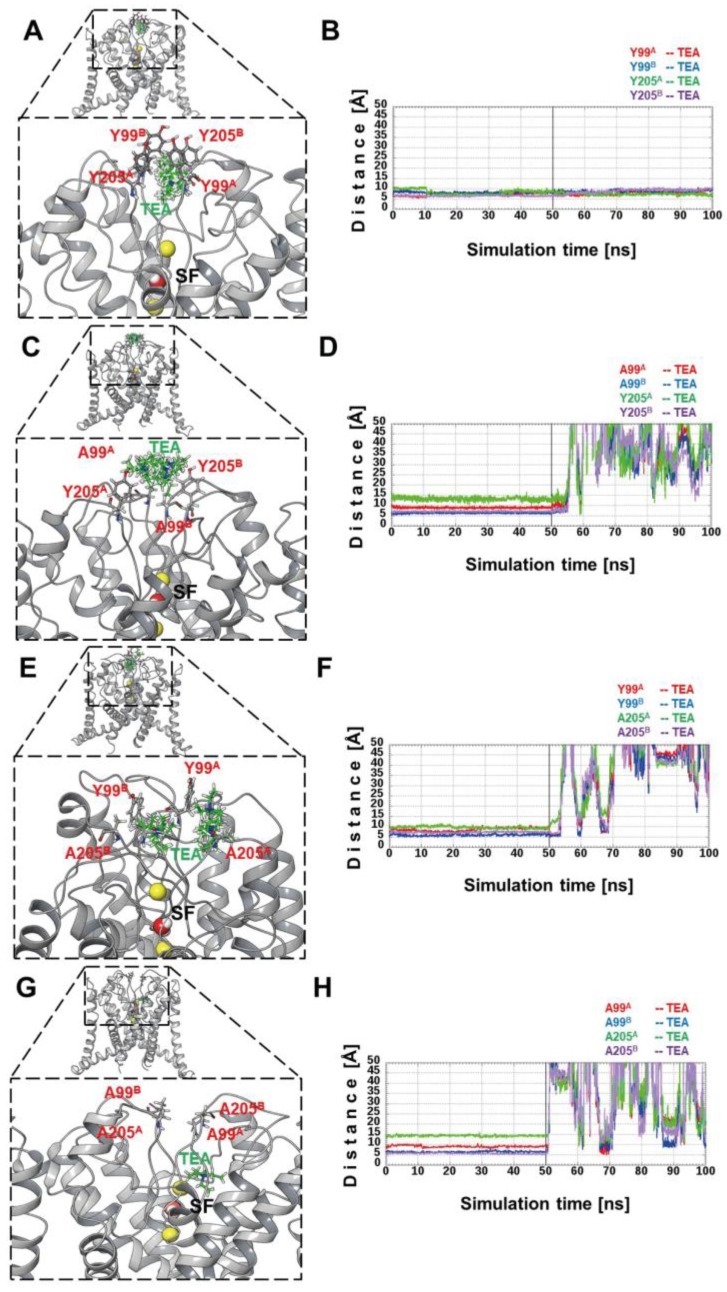
Docking clusters and TEA-Tyr (Tetraethylammonium-Tyrosine) (99 and 205) distances: (**A**,**C**,**E**,**G**) cluster of TEA poses (in green color) obtained by docking analysis for 2loop2, 2loop2/A99, 2loop2/A205 and 2loop2/A99/A205 channels, respectively. In red are shown the residues forming the binding site of TEA near to the selectivity filter (SF). In yellow color, K^+^ ions are depicted and water molecules are represented in red and white, placed in the SF. The superscript letter implies the monomer to which it belongs. (**B**) shows the distances between the 1st best poses and the tyrosine residues, respectively. Similarly, the distances between the 1st best poses and the tyrosine residues 2loop2/A99, 2loop2/A205 and 2loop2/A99/A205 mutant channels are shown in (**D**,**F**,**H**), respectively.

Given that the crystallographic structure for any member of the K_2_P TASK subfamily has not been solved, the best template for TASK-3 was the structure of the TREK-1 channel (Protein Data Bank (PDB) ID code 4TWK), which displays 31% sequence identity and *e*-value = 1E−32. TASK-3/2loop2 and TASK-3 WT models (Figure S3A,B) were subjected to MDs (Molecular Dynamics) by 50 ns. The RMSD (root-mean-square deviation) values for the initial structure of 2loop2 were less than 2 Å (Figure S3C), and continued decreasing gradually with an increase in the simulation time. During the last 12 ns, the RMSD values remain moderately constant, at less than 1 Å. The TASK-3 WT model is 0.2 Å lower than the 2loop2 model until after the first 26 ns, and subsequently the differences were significantly lower. Both models reached an equilibrium in the last 8 ns, approximately. The XP15 (Extra precision) method of Glide docking was used to investigate the binding site of TEA in our models. In the 2loop2 model, only ten poses were found, and all these poses were located in the center of 4 relevant tyrosine residues shown in Figure 6A. 

The cation-Pi interactions found between TEA conformers and Y99 and Y205 residues are in complete agreement with these types of interactions reported for others potassium channels [23,26]. All docking poses were used as a starting point for MM-GBSA (molecular mechanics with generalized Born and surface area solvation) calculations, giving a range ∆GBind between [−41.21: 15.52] kcal mol^−1^ where 80% of these energies were favorable (∆GBind < 0). For the 2loop2/A99/Y205 model depicted in Figure 6C, only seven poses were found, which were ranked between [−2.25: 10.63] kcal mol^−1^ with an 85.7% of unfavorable energies (∆GBind > 0) in TEA poses. In the 2loop2/Y99/A205 mutated channel, seven poses were also found with energies in [−27.20: 1.04] kcal mol^−1^ range (85.7% of poses have favorable energies) (Figure 6E). Nevertheless, four out of seven poses were not in the center of the binding site comprised by the four tyrosine residues (Y99 and Y205), as shown in Figure 6G. Finally, no TEA poses were found in the binding site for 2loop2/A99/A205 mutated channel. However, only one pose was found behind the pocket (shown in Figure 6G) formed by both PD2 in the SF and the N-terminal transmembrane helix 4 (TM4), like the ML335 (*N*-[(2,4-dichlorophenyl)methyl]-4-(methanesulfonamido) benzamide) and ML402 (*N*-[2-(4-chloro-2-methylphenoxy)ethyl]thiophene-2-carboxamide) compounds recently co-crystalized with TREK-1 channel [27]. 

We also investigated the stability of ligand-receptor complexes (obtained by docking methodology) using MDs. Accordingly, the 1st best pose ranked by ∆GBind was subjected to MDs of 100 ns. During the first 50 ns, energy restrains were applied to ligands and the secondary structures of the channels, and during the last 50 ns, the energy restraints over the ligands were removed. To measure the residence time of TEA poses in the binding site, the distance between TEA and the tyrosine (99 and 205, in both monomers) was computed over the whole trajectory. For the 2loop2 channel, its poses remained stables most of the time (Figure 6B), and the first TEA pose lost affinity in the last 4 ns. Given that the distance was calculated using the center of mass of the TEA poses and each tyrosine residue, it is likely that the distance ranges do not correspond to a specific type of interaction but rather only as coordination. For both 2loop2/A99/Y205 and 2loop2/Y99/A205 mutant channels, all TEA poses lose affinity in the binding site before to the first 55 ns, depicted in Figure 6D and F. Because no poses were found in the 2loop2/A99/A205 channel, the best pose of 2loop2 was selected and the four tyrosine residues were mutated to alanine and an energy minimization was applied. Then, the same simulation protocol was applied. As with the other mutant channels (A99/Y205 & Y99/A205), in this case, the TEA pose left the binding site in the first non-restrained ns (Figure 6H).

Taking together, the results shown in Figure 6 confirmed that TASK-3 requires a four-tyrosine ring at the external mouth of the pore for optimal binding to external TEA ions.

## 3. Discussion

The molecular mechanism of blockade of Kv and Kir potassium channels by external TEA has been widely studied [20,21,22,23,26]. These studies have provided relevant insights into the gating and permeation processes of K^+^ channels [20,21,22,23,26]. Regarding K_2_P channels, there is one study in the literature where a detailed study of the blockade of TREK-1 channels by internal TEA was described [28]. On the other hand, K_2_P channels are recognized as extracellular TEA non-sensitive channels [5]. 

The elucidation of the structure of K_2_P channels have provided several clues about the molecular determinants underlying gating processes in K_2_P channels [14,15,16,17]. K_2_P structures revealed that two M1-P1 loops form a cap domain, which has been proposed to form a physical barrier for the access of classical K^+^ channel blockers such as TEA to their binding sites in K_2_P channels [15,16].

In the present article, we used a combination of mutagenesis, functional evaluation and dynamic simulations to challenge the hypothesis that insensitivity of TASK-3 channels for external TEA is due to the presence of the cap structure. Our results suggest that the cap domain in TASK-3 channels effectively restricts the access of extracellular TEA to their binding sites, although the removal of the cap structure does not generate fully blocked TASK-3 mediated K^+^ currents. 

Amino acid sequence analysis of the TASK-3 channel suggested a partial binding site for TEA blocker composed by a tyrosine placed in position 205 at the second P domain. This tyrosine residue resembles the binding site for TEA in Kir and Kv channels, where an aromatic residue (phenylalanine or tyrosine) in position 82 or 320 (KcsA or Kv1.2 channel, respectively, see Figure 2A) play an essential role in TEA binding [20,21,22,23]. Given the tetrameric architecture of Kir and Kv channels, the presence of a tyrosine generates a four-tyrosine ring to TEA coordinate via π-cation interaction [22,26]. 

If only four aromatic residues are responsible for TEA binding in other K^+^ channels, we hypothesized that engineering a ring composed of four tyrosine residues might result in TASK-3 channels highly sensitive for TEA ions. As proof of concept, we introduced an extra tyrosine residue either in position 99 (A99Y) or 100 (A100Y) in TASK-3 channel and assessed the sensitivity of this channel to TEA. Our results showed that TASK-3 channels are partially blocked by TEA ions when four tyrosine residues were placed near the pore region. Strikingly, the A99Y mutant was fully sensitive to external TEA ions when the cap structure was removed from TASK-3 channels.

Functional analysis of the cap-less construct (TASK-3 2loop2) displayed a TEA sensitivity with an IC_50_ close to that obtained for Kv2.1 channel. Our results provide strong evidence supporting for residues Y99 and Y205 forming part of the binding site for TEA: Y99A and Y205A mutants resulted in cap-less TASK-3 channels with partial sensitivity to external TEA ions (Figure 5C–F). Additionally, the double mutant 2loop2/Y99A/Y205A a substantial reduction in the sensitivity to TEA. Taken together, our results show support for Y205 as part of a TEA-binding site in TASK-3 channels. Moreover, the mutants 2loop2/Y99A/Y205A in TASK-3 (dose-response shown in Figure 5H) and Y380A in Kv2.1 channel (dose-response shown in Figure S1) still showed some sensitivity to TEA ions, suggesting that other residues from both channels located in the K^+^ permeation pathway might be important for TEA binding. More experiments in the future are required to evaluate the contribution of other residues to TEA binding.

The cap structure deletion generated in the construct TASK-3/2loop2 also evidenced the relevance of extracellular ion pathway (EIP) for TASK-3 channel function. Functional evaluation of TASK-3/2loop2 showed a loss of K^+^ selectivity. This loss of selectivity displayed by cap-less TASK-3 channels might be due to a constitutive C-type inactivation caused by the absence of the cap structure [29,30,31,32]. In this case, the cap structure might be acting as a K^+^ concentrative pathway near to the pore region and its removal could be associated with lower local K^+^ concentrations near the pore that may result in a pore collapse. The robust activity of cap-less TASK-3 channels recorded under symmetrical high K^+^ concentrations are in agreement with this hypothesis, although further experiments are required in order to confirm the mechanism underlying loss of selectivity in the cap-less channels.

According to our homology model of TASK-3, the EIP of the cap structure has a group of amino acids that generate an electronegative potential (Q68, E70, P71, G75, Q77 and H98), which could increase the concentration of potassium in the extracellular conduction pathway [18]. Our model generated for the cap-less TASK-3 is consistent with a decreased electronegative potential and with the consequent effect on the selectivity filter, which was confirmed when the electrostatic potential was evaluated for the WT and TASK-3/2loop2 models (Figure S4). In contrast to other K_2_P channels, the cap deletion did not affect the expression or dimerization of TASK-3 channel, ruling out an essential role of the cap in the dimerization of TASK-3 channels.

In conclusion, our study revealed that cap structure explains, at least in part, the poor sensitivity of K_2_P channels to TEA. Moreover, the cap structure is not essential to the channel expression or assembly. Our data also supports for a key role of the cap structure in TASK-3 channel function by maintaining the architecture in the mouth of the pore.

## 4. Materials and Methods 

### 4.1. Constructs

*Cavia porcellus* TASK-3 (GenBank accession No AF212827) was obtained from Dr. Jürgen Daut (Marburg University, Marburg, Germany). *Rattus norvegicus* Kv2.1 (GenBank under accession No NM_013186) cDNA was subcloned into pMAX (eukaryotic expression vector) vector and provided by Dr. Steve Goldstein (Loyola University Chicago, Chicago, IL, USA). Mutants and deletion constructs were generated by PCR (Taq DNA polymerase, Thermo Scientific, Waltham, MA, USA) using standard protocols. The sequences of amplified regions were confirmed by DNA sequencing.

### 4.2. Electrophysiological Recordings

HEK-293 cells were maintained in DMEM-F12 media (Invitrogen Life Technologies, Carlsbad, CA, USA) supplemented with 10% FBS and 1% penicillin/streptomycin. Plasmid transient transfections (1–2 µg plasmid) were done with a DNA ratio of 3:1 (plasmid encoding channel: plasmid encoding for GFP as marker) using Xfect polymer (Clontech, Mountain View, CA, USA). Whole cell recordings were performed at room temperature for 24 to 48 h. post-transfection using a PC-501A patch clamp amplifier (Warner Instruments, Hamden, CT, USA) and borosilicate pipettes as described elsewhere [29]. Cells were continuously perfused with bath solution containing (in mM): 135 NaCl, 5 KCl, 1 MgCl_2_, 1 CaCl_2_, 10 HEPES, 10 Sucrose, adjusted to pH 7.4 with NaOH. Intracellular pipette solution contained (in mM): 145 KCl, 5 EGTA, 2 MgCl_2_, 10 HEPES, adjusted to pH 7.4 with KOH. External high K^+^ solution was obtained by equimolar substitution of Na^+^ by K^+^. Tetraethylammonium chloride (Sigma-Aldrich, St. Louis, MO, USA) was directly dissolved in external bath solutions to obtain the desired final concentrations. Control experiments designed to rule out a possible contribution of external osmolality were performed using the bath solution described above but supplemented with D-Mannitol.

### 4.3. Homology Modeling

Five different models for TASK-3 and its variants were built by homology using the structure of the TREK-1 channel (PDB: 4TWK) as a template using the software MODELLER (University of California, San Francisco, CA, USA) [33]. Both monomers were optimized by Molecular Dynamics (MD) and evaluated using Energy (DOPE) [34] and Procheck programs. The models were prepared in Maestro suite and protonation states were assigned with PROPKA software at pH 7.4. The structures were refined by means of energy minimization in vacuum with a conjugate gradient algorithm. Afterward, the models were embedded into a pre-equilibrated POPC (phosphatidylcholine) bilayer and solvated in a cubic box with SPC (simple point charge model) water molecules, in periodic boundary conditions adding 150 mM of NaCl. Subsequently, the system was relaxed by MDs for 50 ns with 0.25 kcal mol^−1^ Å^−2^ of harmonic energy restraints, applied to the secondary structure (excepting loops), using a Desmond package and OPLS software (Desmond Molecular Dynamics System, New York, NY, USA) [35]. To replicate the thermodynamic condition in wet-lab, isothermal-isobaric (NPT) ensembles at 1.01325 bar and 300 K method as thermostat were used. Root-mean square deviations (RMSD) were computed over all heavy atoms along the MD trajectory to evaluate equilibrium convergence.

### 4.4. Computational Mutagenesis (CM)

The construct TASK-3/2loop2, that displays no cap structure and two loop2-P2 per subunit, has 4 tyrosine residues: a Y99 and Y205 from each monomer. These residues are positioned in the extracellular mouth of channel just above to the selectivity filter (SF), in direct contact with the aqueous medium. Tyrosines 99 and 205 were subjected to CM. The last structure from the MD trajectory of TASK-3/2loop2 was used as starting point to CM, according to the scheme represented in Table 1. All mutations were performed with Maestro suite and then all residues within 8 Å of cutoff from the mutated residue were subjected to energy minimization in implicit solvent.

### 4.5. Docking and Molecular Mechanics Energies Combined with Generalized Born and Surface Area Continuum Solvation (MM-GBSA) Studies

TEA structure was downloaded from PDB (ID: 1T36) in SDF (Spatial Data File) format and then prepared with Ligprep tool in OLPS (Optimized Potential for Liquid Simulations) 2005. All possible protonation states for TEA at physiological pH were generated using the Epik program [36]. To assess the binding site of TEA in our channel models, docking studies were carried out in all systems shown in Table 1. Before docking, the K^+^ ion located in the first site (S1) of SF was removed to avoid the TEA-ion electrostatic repulsion. The conformational search of TEA was carried out in a grid box placed in the extracellular portal of the channel, using the geometric coordinates of S1 and dimensions of 26 × 26 × 26 Å in each edge of the box. The Extra Precision (XP) algorithm of Glide, flexible ligand sampling and default docking parameters were used [37]. Docking assays were followed by MM-GBSA method to obtain the relative binding affinities of docking conformers. The MM-GBSA energies were computed over all docking outputs using OPLS 2005 and Prime program. The protein was subjected to an energy minimization within 8 Å of radius from the ligand. Subsequently, al No, it means potassium binding site number 1l conformers in each system were ranked by the relative binding affinities (∆GBind) values.

### 4.6. Molecular Dynamics Simulations (MDs)

The conformers for each system shown in Table 1 were ranked by ∆GBind and subjected to MDs (100 ns). For the first 50 ns of simulation, an energy restraint of 0.5 kcal mol^−1^ Å^−2^ was applied to the ligands, which allows channels to adapt to the ligand. Then, the energy restraints over the ligands were removed after the first 50 ns. During the whole simulation time, energy restraints were applied to secondary structure of the channel (0.25 kcal mol^−1^ Å^−2^). To evaluate the coordination and the time of residence of TEA within the binding site for all systems, the distances between the TEA mass center and the mass center of each residue (numbers 99 and 205, in both monomers) during 100 ns were measured. The electrostatic potential surfaces were computed with APBS20,21 v1.4 over the protein as a mean in whole simulation time (supporting material S4).

### 4.7. Statistical Analysis

Data were compiled and analyzed with the SPSS software package, version 17.0 (SPSS Inc., Chicago, IL, USA). Individual experimental TEA blockade data were fitted to a four-parameter logistic function, described by the following equation:

*I*/*I_max_* = (*I_min_* − *I_max_*)/{[1 + ([TEA]/IC_50_)*^h^*] + *I_min_*} (1)

where *I*/*I_max_* is the blocked fraction of K^+^-mediated currents. *I_min_* and *I_max_* represent minimal and maximal currents, and *h* and IC_50_, represent the Hill coefficient and concentration of TEA producing half-maximal inhibition of TASK-3 currents, respectively. Significance of differences between means were calculated with unpaired Student’s *t* test. All data shown are mean ± standard error of mean (SEM).

## Figures and Tables

**Table 1:** Description for the four mutants used during the study.

System Name	Description Per Subunit	Mutant Type	Tyrosine in Both Subunit
2loop2	1 Y in 1st PD1 1 Y in 2nd PD2	-	4 (2 per monomer)
2loop2/Y99A	1 A in 1st PD1 1 Y in 2nd PD2	Y99A	2 (1 per monomer)
2loop2/Y205A	1 Y in 1st PD1 1 A in 2nd PD2	- Y205A	2 (1 per monomer)
2loop2/Y99A/Y205A	1 A in 1st PD1 1 A in 2nd PD2	Y99A Y205A	0 (0 per monomer)

In construct named 2loop2, the cap structure from TASK-3 channels was removed and the 1st Pore Domain (PD1) was replaced by 2nd Pore Domain (PD2). Hence, 2loop2 has two PD2 with 2 Tyr (Tyrosine) residues (Y99 & Y205) per subunit, forming the putative binding site of TEA. In the mutant 2loop2/Y99A, the Y99 was mutated to alanine in both subunits (Y99A^A^, Y99A^B^). In 2loop2/Y205A, the Y205 was mutated to alanine and 2loop2/Y99A/Y205A, theY99 and Y205 were mutated by alanine in both subunits, generating a channel without a binding site for TEA.

## Supplementary Materials

Supplementary materials can be found at http://www.mdpi.com/1422-0067/19/8/2437/s1.
