# Correction: Concha, G., et al. The Insensitivity of TASK-3 K_2_P Channels to External Tetraethylammonium (TEA) Partially Depends on the Cap Structure. *Int. J. Mol. Sci.* 2018, *19*, 2437

**DOI:** 10.3390/ijms20184651

**Published:** 2019-09-19

**Authors:** Guierdy Concha, Daniel Bustos, Rafael Zúñiga, Marcelo A. Catalán, Leandro Zúñiga

**Affiliations:** 1 Centro de Investigaciones Médicas (CIM), Programa de Investigación Asociativa en Cáncer Gástrico (PIA-CG), Escuela de Medicina, Universidad de Talca, Talca 3460000, Chile; guierdy@gmail.com (G.C.); danielbustos.ibi@gmail.com (D.B.); rafaelzunigah@gmail.com (R.Z.); 2 Laboratorio de Fisiología Epitelial, Facultad de Ciencias de la Salud, Universidad Arturo Prat, Iquique 1130000, Chile; macatalan@unap.cl; 3 Magíster en Gestión de Operaciones, Facultad de Ingeniería (Campus Los Niches), Universidad de Talca, Talca 3460000, Chile; 4 CBSM y Doctorado en Ciencias, mención Modelado de Sistemas Químicos y Biológicos, Facultad de Ingeniería, Universidad de Talca, Talca 3460000, Chile

We would like to submit the following correction to our published paper [1]: The following affiliations and acknowledgments should be included.

**Guierdy Concha ^3^ and** **Daniel Bustos ^4^**

^3^  Magíster en Gestión de Operaciones, Facultad de Ingeniería (Campus Los Niches), Universidad de Talca, Talca 3460000, Chile
^4^  CBSM y Doctorado en Ciencias, mención Modelado de Sistemas Químicos y Biológicos, Facultad de Ingeniería, Universidad de Talca, Talca 3460000, Chile

**Acknowledgments:** Proyecto Fondecyt 1140624 (Wendy González) of the Fondo Nacional de Ciencia y Tecnología (Chile).

These changes do not change the results and conclusions of our paper. We apologize for any inconvenience caused.
