# Supplementary material for: The Insensitivity of TASK-3 K2P Channels to External Tetraethylammonium (TEA) Partially Depends on the Cap Structure
Source: Int J Mol Sci. 2018 Aug 18;19(8):2437. doi: 10.3390/ijms19082437 (PMC6121469; doi:10.3390/ijms19082437)
Supplement: Supplementary file 1 [file ijms-19-02437-s001.pdf]

International Journal of
Molecular Sciences

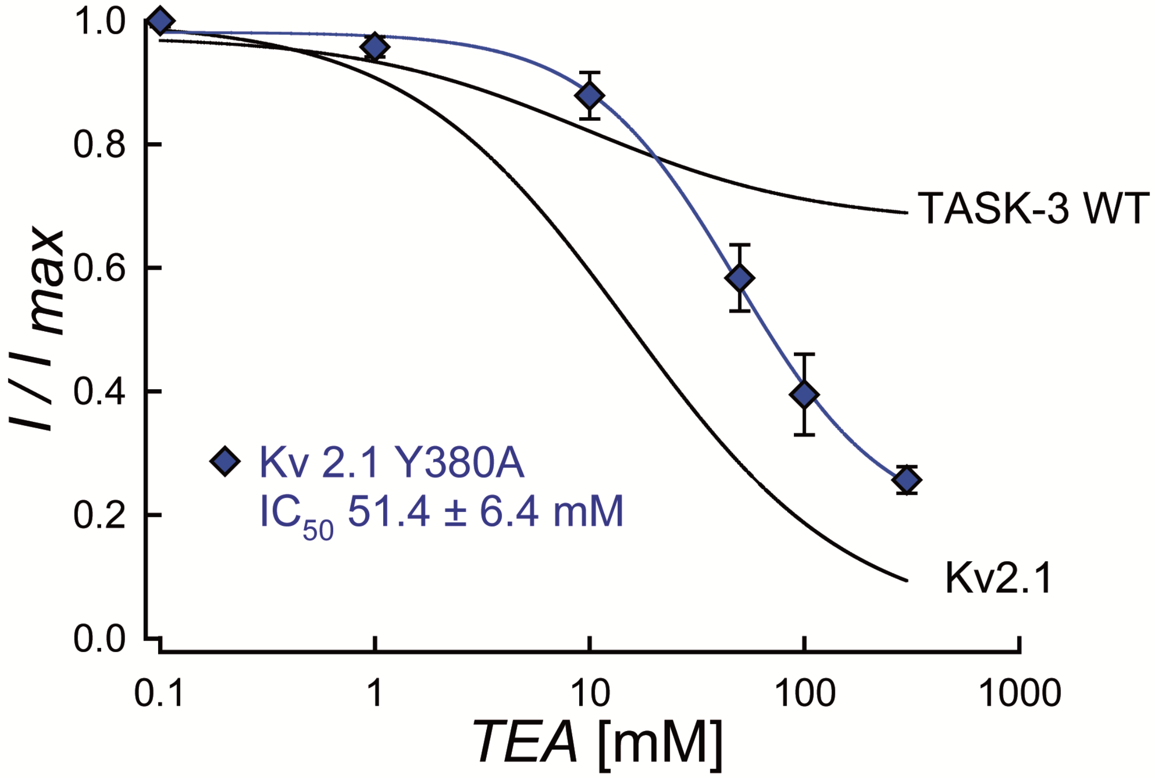

Figure S1. Effect of TEA on Kv2.1/Y380A mutant channels expressed in HEK-293 cells. Dose-response curve of TEA on Y380A mutant (blue diamonds, $n = 3$). Channel blockade was analyzed at the end of the test pulse to +80 mV. Results are shown as means ± SEM. The black lines (without symbols) are taken from the fits in Figure 1C and correspond to the TEA inhibition curves for TASK-3 WT and Kv2.1, respectively.

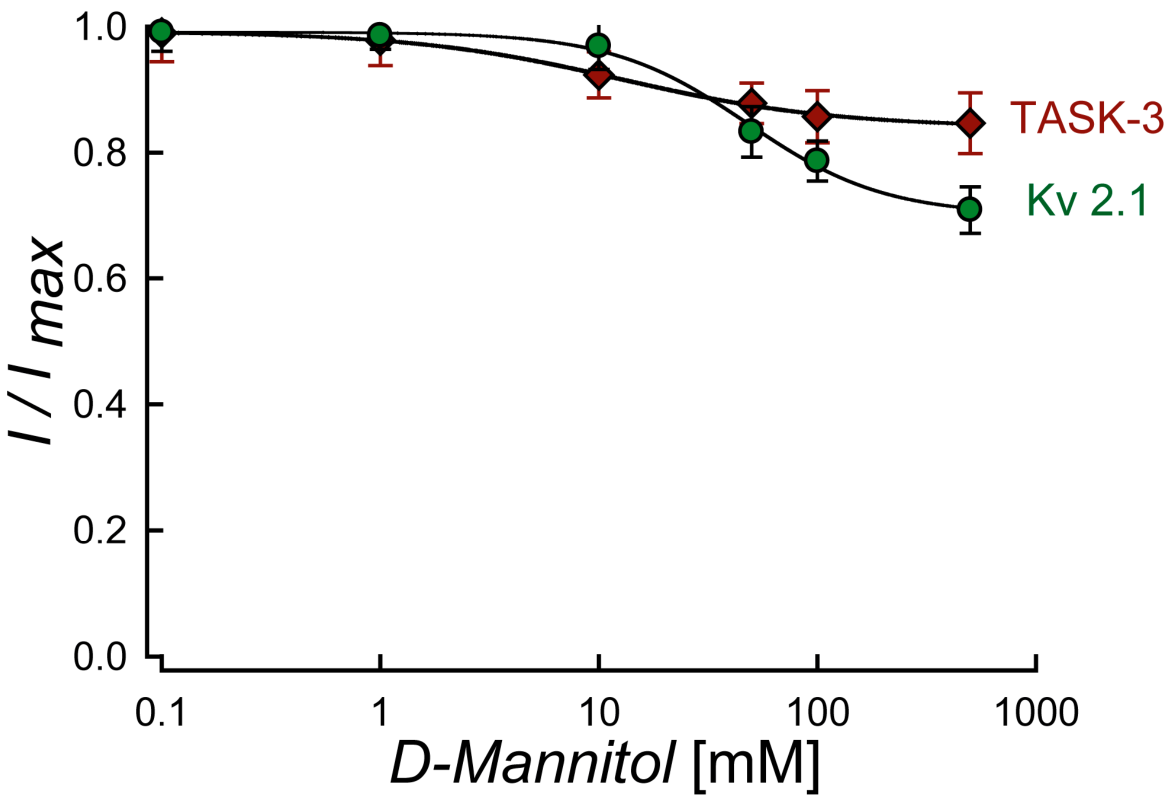

Figure S2. Effect of hypertonicity on TASK-3 and Kv2.1 currents expressed in HEK-293 cells. The graph shows the hypertonicity-dependence curves displayed by TASK-3 (diamonds, *n* = 4) and Kv2.1 (circles, *n* = 3). Results are shown as means ± SEM. The changes in tonicity of the extracellular solution from 300 (isotonicity) to 800 (hypertonicity) mOsm achieved by the addition of D-Mannitol.

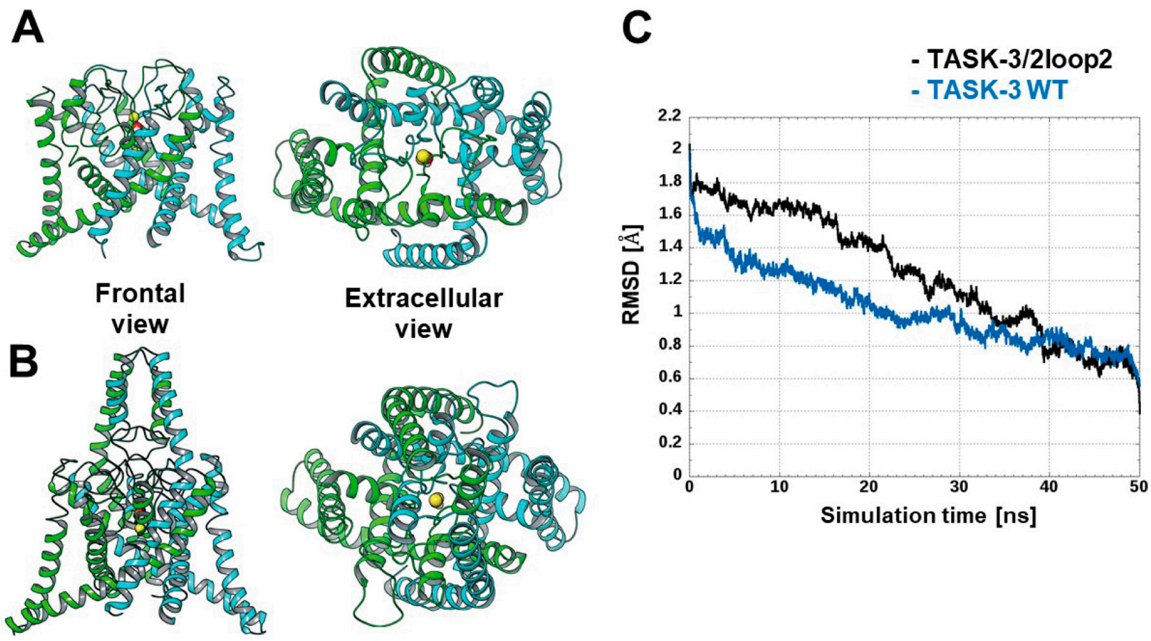

Figure S3. Molecular models obtained for TASK-3 channels: (**A**) Frontal (left side) and external (right side) views of 2loop2 model. (**B**) frontal (left side) and external (right side) views of TASK-3 WT model. Subunits are coloured in green (monomer A) and cyan (monomer B). K^+ ions located in the selectivity filter (SF) are shown in yellow colour, water molecules are shown in white and red colour. (**C**) Root Mean Square Deviation calculated to all the heavy atoms during 50 ns for 2loop2 (in black colour) and WT (in blue colour) models.

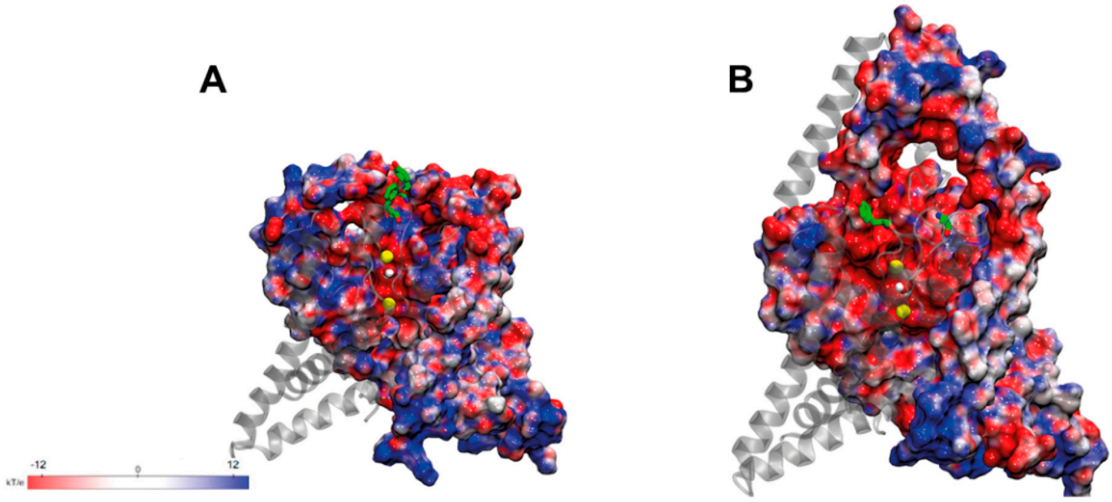

Figure S4. Electrostatic Potential Surfaces (EPS) of TASK-3 models. (**A**) 2loop2 and (**B**) TASK-3 WT channels computed as a mean over 50 ns of MDs. K^+ ions located in the SF are shonw in yellow colour, water molecules are shown in white and red colour. The EPS is displayed in monomer A while monomer B is depicted in silver colour. Negative, positive and neutral regions of EPS are depicted in red, blue and white colours, respectively. Besides, residues 99 and 205 are displayed in green colour and correspond to those residues from monomer B. Note that the residues 99 and 205 correspond to tyrosine residues positioned in 1st PD2 and 2nd PD2 in 2loop2/A99Y channel while positions 99 and 205 in TASK-3 WT channels correspond to alanine and tyrosine residues positioned in PD1 and PD2, respectively.

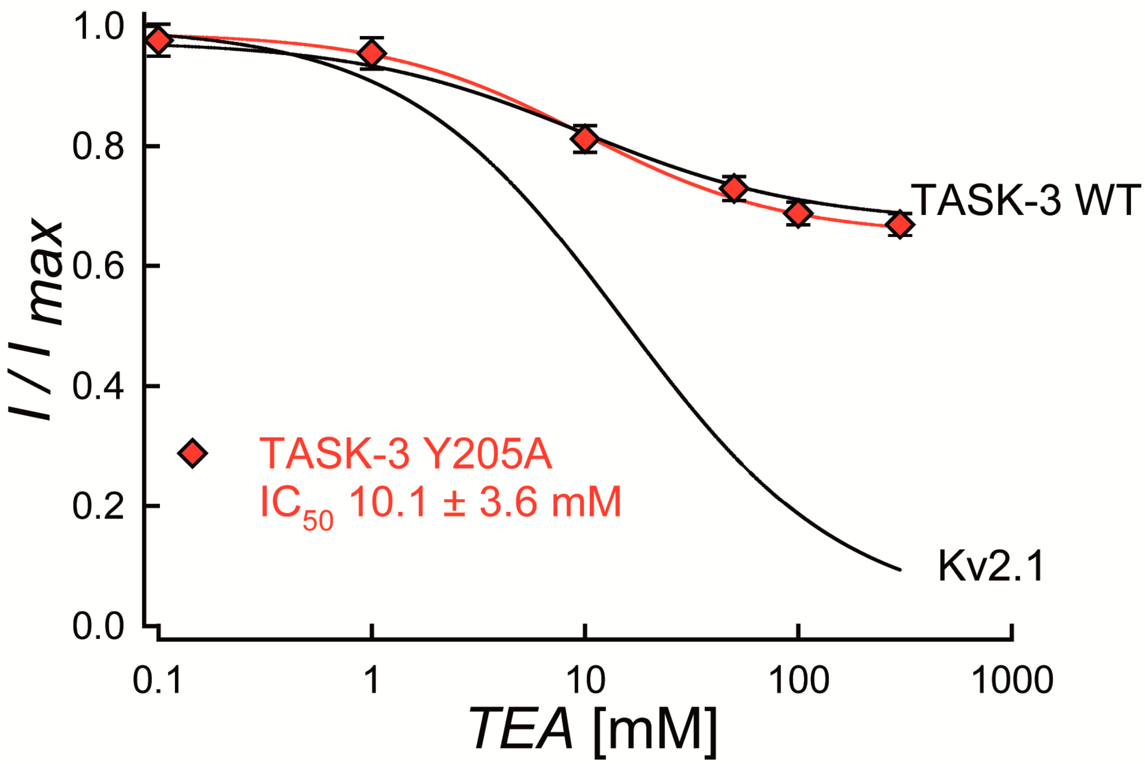

Figure S5. Effect of TEA on TASK-3/Y205A mutant channels expressed in HEK-293 cells. Dose-response curves for the blockade effect mediated by TEA on TASK-3/Y205A mutant (red diamonds, $n = 6$). Blockade was analyzed at the end of the test pulse to +80 mV. Results are shown as means ± SEM. The solid lines (with no symbols) are taken from the fits shown in Figure 1C and correspond to the TEA inhibition curves for TASK-3 WT and Kv2.1, respectively.
